# Biotechnological and Therapeutic Application of Useful Plants in Endocrinal Disorder

**DOI:** 10.1155/2017/3140683

**Published:** 2017-10-12

**Authors:** Na-Hyung Kim, Vishal Chandra, Shruti Shukla, Pradeep Kumar

**Affiliations:** ^1^Department of Oriental Pharmacy, Wonkwang University, Iksan 02404, Republic of Korea; ^2^Department of Obstetrics and Gynecology, Stephenson Cancer Center (SCC), University of Oklahoma Health Sciences Center (OUHSC), Oklahoma City, OK, USA; ^3^Department of Energy and Materials Engineering, Dongguk University, Seoul 100-715, Republic of Korea; ^4^School of Biotechnology, Yeungnam University, Gyeongsan, Gyeongbuk, Republic of Korea

The use of natural products in the remedies for health care and various diseases or traditional medicine has been around for a long time in many countries. Recent advances in the fields of natural products including various plants have been widely utilized in an aspect of biotechnology together with therapeutic applications. The active constitutes isolated or characterized from natural products have been developed as the lead ingredients of novel pharmaceuticals, alternative medicines, and nutraceuticals. Consistent efforts are being paid on treating metabolic diseases, cardiovascular diseases, carcinoma, inflammatory diseases, and infection around the world using natural products. In the current issue, we will deal with recent investigations of endocrinal disorder related to a network of glands which produce or release hormones that regulate in cell and organ of body. Endocrinal disorder in human can be induced by alterations of genetic factors and the exposure to endocrine disrupting chemicals as environmental factors has an impact on human life. We hope that biotechnological and therapeutic application of useful plants and their active constitutes may have efficacy to be developed as alternative remedies in curing endocrinal disorder in human disease.

For this special issue we invited global investigators to submit their original research and review articles involving biotechnological and therapeutic application using useful plants of natural products for controlling endocrine diseases including obesity/diabetes/hyperlipidemia related to metabolic syndrome, osteoporosis related to bone, thyroid disease, cardiovascular, or renal diseases, and hormonal growth disorders. In addition, we also invited the articles that investigate physiologic effects of the plants, its composition, and nutrients, using both* in vitro* and* in vivo* animal models, together with clinical trials. After a rigorous peer-review process, various studies on biotechnological and therapeutic application using useful plants of natural products for controlling endocrine diseases were accepted as the articles in this special issue.

Goiter, an enlargement of the thyroid gland, was reported which can be associated with a number of thyroid diseases such as thyroid dysfunction (hyperthyroidism and hypothyroidism), autoimmune thyroid disease (Graves' disease and Hashimoto's thyroiditis), thyroiditis, and thyroid cancer. L. Xiu et al. showed that HYDf and HYDp, which are two different species of Haizao Yuhu Decoction (HYD), have been widely used to treat thyroid-related diseases especially goiter with few side effects in traditional Chinese medicine (TCM), including herb pair* Sargassum* (HZ) and Glycyrrhizae Radix et Rhizoma (GC), could exhibit antigoitrous effect through alterations in hypothalamus-pituitary-thyroid (HPT) axis and inhibition of the TPO gene expression in goiter rat model.

Endometriosis is reported as a common benign disorder characterized by the ectopic growth of endometrium, affecting approximately 15% of women of reproductive age, causing chronic pelvic pain, dysmenorrheal, irregular menstrual cycle, and infertility. R.-N. Liang et al. demonstrated that Ping-Chong-Jiang-Ni formula (PCJNF), a Chinese herbal medicine (CHM), was shown to be clinically effective on endometriosis. They explored the effect of PCJNF on the ectopic endometrial stromal cells (EESCs) from endometriosis. PCJNF could suppress cell proliferation, migration, and invasion, while increasing apoptosis in EESCs, and the suppressed proliferation and enhanced apoptosis were mediated by JNK signaling.

X. Wang et al. demonstrated that compounds from Cynomorium songaricum (CS) exhibited phytoestrogenic and phytoandrogenic activities using MCF-7 or LNCaP cells and HeLa or AD293 cells, which may contribute to inhibiting the oestrogen/androgen-induced BPH development. Y. Zhang et al. showed that Pingmu Decoction could reduce orbital preadipocytes viability, induce apoptosis of mature adipocyte via Fas/Fas L signaling pathway, and reduce lipid accumulation and downregulated the expression of PPAR *γ* and C/EBP *α* in preadipocytes of Graves' Ophthalmopathy (GO) patients from differentiating into mature adipocytes and medicinal serum which was prepared from rats.

In an aspect of osteoporosis, S.-W. Choi et al. demonstrated that barley seedling extracts (BSE) dose-dependently inhibited RANKL-induced osteoclast differentiation with alteration of I*κ*B degradation, c-Fos, and NFATc1 molecules in the early-to-middle stages of osteoclastogenesis. Additionally, in the late phase of osteoclastogenesis, BSE also prevented DC-STAMP and cathepsin K, which are required for cell fusion and bone degradation, such as osteoclast function.

A variety of studies in this special issues could provide new insights to treat and prevent endocrinal disorders.

